# New dynamic suture material for tendon transfer surgeries in the upper extremity – a biomechanical comparative analysis

**DOI:** 10.1007/s00402-024-05322-5

**Published:** 2024-05-02

**Authors:** Tatjana Pastor, Ivan Zderic, Mehar Dhillon, Boyko Gueorguiev, R. Geoff Richards, Torsten Pastor, Esther Vögelin

**Affiliations:** 1 AO Research Institute Davos, Davos, 7270 Switzerland; 2 Department for Plastic and Hand Surgery, Inselspital University Hospital Bern, University of Bern, Bern, Switzerland; 3 Department of Orthopaedic and Trauma Surgery, Lucerne Cantonal Hospital, Lucerne, Switzerland

**Keywords:** Tendon transfer, FiberWire, Dynacord, Tendon approximation, Early rehabilitation, Biomechanics

## Abstract

**Background:**

Early mobilization after tendon surgery is crucial to avoid commonly observed postoperative soft tissue adhesions. Recently, a new suture was introduced (DYNACORD; DC) with a salt-infused silicone core designed to minimize laxity and preserve consistent tissue approximation in order to avoid gap formation and allow early mobilization.

**Aims:**

To compare the biomechanical competence of DC against a conventional high strength suture (FiberWire; FW) in a human cadaveric tendon transfer model with an early rehabilitation protocol.

**Methods:**

Sixteen tendon transfers (flexor digitorum superficialis (FDS) IV to flexor pollicis longus (FPL)) were performed in 8 pairs human cadaveric forearms using either DC or FW. Markings were set 0.8 cm proximally and 0.7 cm distally to the level of the interweaving zone of the transfer. All specimens underwent repetitive thumb flexion against resistance in 9 intermittent series of 300 cycles each, simulating an aggressive postoperative rehabilitation protocol. After each series, the distance of the proximal marker to the interweaving zone (proximal), the length of the interweaving zone (intermediate) and the distance of the distal marker to the interweaving zone (distal) were measured.

**Results:**

Pooled data over all nine series, normalized to the immediate postoperative status, demonstrated no significant differences between FW and DC (*p* ≥ 0.355) for the proximal and distal markers. However, at the intermediate zone, DC was associated with significant length shortening (*p* < 0.001) compared to FW without significant length changes (*p* = 0.351). Load to catastrophic failure demonstrated significant higher forces in FW (*p* = 0.011). Nevertheless, due to failure mainly proximal or distal of the transfer zone, these loads are not informative.

**Conclusion:**

From a biomechanical perspective, DC preserved tissue approximation and might be considered as a valid alternative to conventional high-strength sutures in tendon transfer surgery. DC might allow for a shorter interweaving zone and a more aggressive early postoperative rehabilitation program, possibly avoiding commonly observed postoperative soft tissue adhesions and stiffness.

**Supplementary Information:**

The online version contains supplementary material available at 10.1007/s00402-024-05322-5.

## Introduction

There are different reasons for required tendon reconstruction or transfers in the upper extremity i.e. after paralysis of muscle groups caused by nerve lesions, long-lasting nerve compression syndromes, spinal cord injury or cerebral palsy, and tendon defects due to trauma or infection. Other reasons include tendon ruptures due to chronic irritation in inflammatory diseases such as rheumatoid arthritis [1, 2]. Furthermore, chronic tendon weakening and rupture due to conflict with hardware after fracture fixation (too long screws, position of protruding plate) may damage tendons in long term [3]. In cases of volar plating of distal radius fractures, a secondary rupture of the flexor pollicis longus (FPL) tendon occurs in up to 12% at 10 months on average after surgery [4–6]. In such cases, a primary tendon repair is not possible due to the degenerated tendon quality and tendon transfers are required. Therefore, either the superficial flexor tendon (FDS) of the ring or the middle finger is transferred to the FPL tendon stump of the thumb for reconstruction [7]. After a short interval between rupture of the FPL and surgery, a tendon reconstruction with a tendon graft between the proximal FPL tendomuscular origin and the distal FPL tendon may be alternatively used [8]. After tendon transfer or tendon reconstruction, patients’ finger movements are sometimes restricted for several weeks in order to avoid gap formation between the sutured tendons jeopardizing their healing [9]. There is no consensus in the literature regarding the optimal postoperative protocol and the duration of immobilization, and the timing of rehabilitation varies among different studies [9–11]. However, prolonged postoperative immobilization causes adhesions of the soft tissues leading to impaired tendon function [9, 12]. Since tendon healing cannot be accelerated, a balance between strong suture material without causing foreign body reaction or adhesions and early active mobilization (immediate postoperative active finger mobilization) must be defined. Recently, a new suture material (DYNACORD Suture (DC), DePuy Synthes Mitek Sports Medicine, Chesterfield, MA, USA) has been introduced, designed to minimize the laxity after tendon repair and provide consistent compression between the sutured structures. It features a silicone core interspersed with salt, attracting water. In a liquid environment, such as in the human body, fluid absorption leads to radial expansion of the suture material due to swelling of its core, resulting in shortening of the braid and thus a self-tensioning of the suture material. Yet, this new suture material has not been subjected to a direct biomechanical evaluation in tendon transfer surgery so far. Therefore, the aim of the current study was to investigate the biomechanical competence of DC against a conventional high-strength suture FiberWire ((FW), Arthrex, Naples, FL, USA) in a human cadaveric tendon transfer model under an early aggressive rehabilitation protocol. It is hypothesized that DC maintains the tissue approximation during early finger movement, possibly allowing for a more active postoperative aftercare to prevent soft tissue adhesions.

## Materials and methods

### Specimens and study groups

Eight pairs of fresh-frozen human cadaveric forearms from 4 male and 4 female donors aged 72.5 years on average (range 48–96 years) were used. The specimens were obtained from Science Care (Phoenix, AZ, USA), following approval of the donors for use of their bodies in medical science during their lifetime. All experiments were carried out under the relevant guidelines and regulations. The forearms underwent screening to exclude prior injuries to the bone, joints, and soft tissue. Further exclusion criteria were chronic issues, such as rheumatological and neurological disorders due to concomitant impairment of joint mobility. All specimens were thawed at room temperature for 24 h prior to preparation and assigned to two groups for treatment with either DC or FW, with equal distribution of left and right anatomical sites in each group.

### Surgical technique

**Fig. 1:**
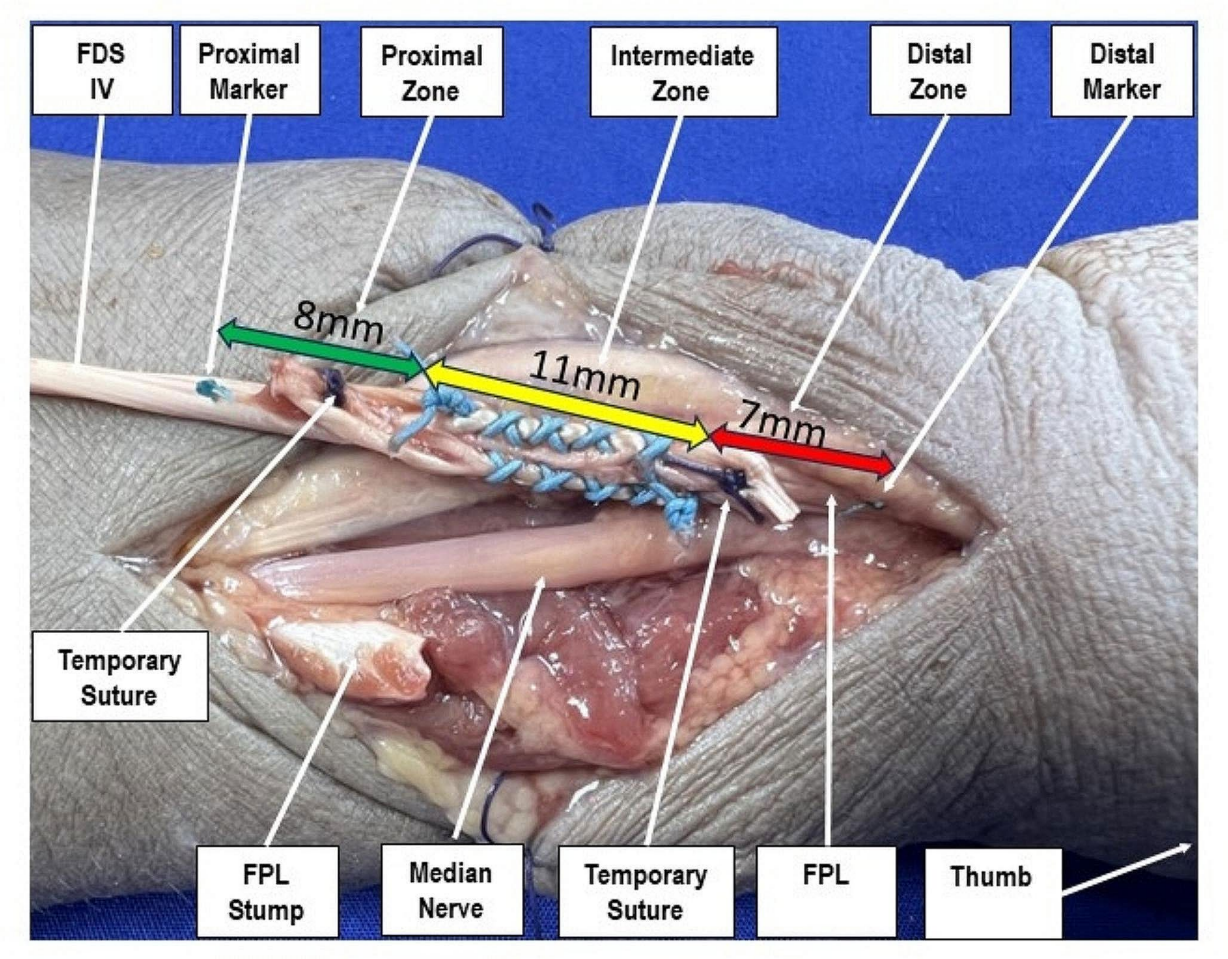
Exemplified photograph of the side-to-side transfer with DC suture material—view from volar to a right specimen. FDS: superficial flexor tendon; FPL: flexor pollicis longus; green arrow: proximal zone; yellow arrow: intermediate zone; red arrow distal zone. The temporary sutures were removed prior to testing

FDS-IV to FPL transfer surgery was performed in all specimens at the level of the distal forearm using a standard approach to the distal wrist according to the modified palmar (Henry) approach (Fig. 1). A second 2.5 cm incision in the palm of the hand in extension of the 4^th^ digit for decompression of the carpal tunnel was performed. After that, a blunt dissection up to the palmar aponeurosis was performed, and the retinaculum flexorum was cut at its ulnar edge. Furthermore, the distal portions of the forearm fascia were split. Subsequently, a Bruner incision was performed in the palm of the hand above the metacarpophalangeal (MCP) joint. The FDS-IV tendon was isolated and dissected between the A1 and A2 pulley under wrist and ring finger flexion. After passing the FDS-IV tendon proximally dorsal to the median nerve to the distal forearm, it could be weaved into the FPL tendon, which was dissected 7 cm proximal to the base of the first metacarpal bone. After checking the correct pretension, a temporary fixation of the interweaving of the two tendons was made with PDS 3-0 sutures (Ethicon, Cincinnati, OH, USA) to assist during final suturing. Subsequently, fixation of the side-to-side suture according to Fridén [13] was performed either with DC or FW using 4 surgeons’ knots to secure the suture in DC and 7 in FW, which is the minimum number of throws to achieve a secure knot with both suture materials according to van Knegsel et al. [14]. Due to technical considerations and in contrast to Fridén’s technique [13], a length of 11 mm interweaving zone was chosen in the current study.

Next, a permanent mark was set by means of a PDS 3-0 suture 0.8 cm proximal and 0.7 cm distal to the side-to-side suture, acting as landmark during measurement after biomechanical testing. Based on these markings, the sutured tendon was divided in 3 zones: proximal—from the start of the suture with either DC or FW to the proximal marker, distal—from the end of the suture with either DC or FW to the distal marker, and intermediate—11 mm interleaving zone of DC or FW (Fig. 1). Finally, the FDS-IV tendon was cut at its musculo-tendinous transition and attached with a PDS 3-0 suture to a looped 1.5 mm braided steel wire. Fingers II-IV were taped in extension in order to allow free passage of the flexing thumb. All instrumentations and measures were performed by one experienced hand surgeon.

### Biomechanical testing

**Fig. 2:**
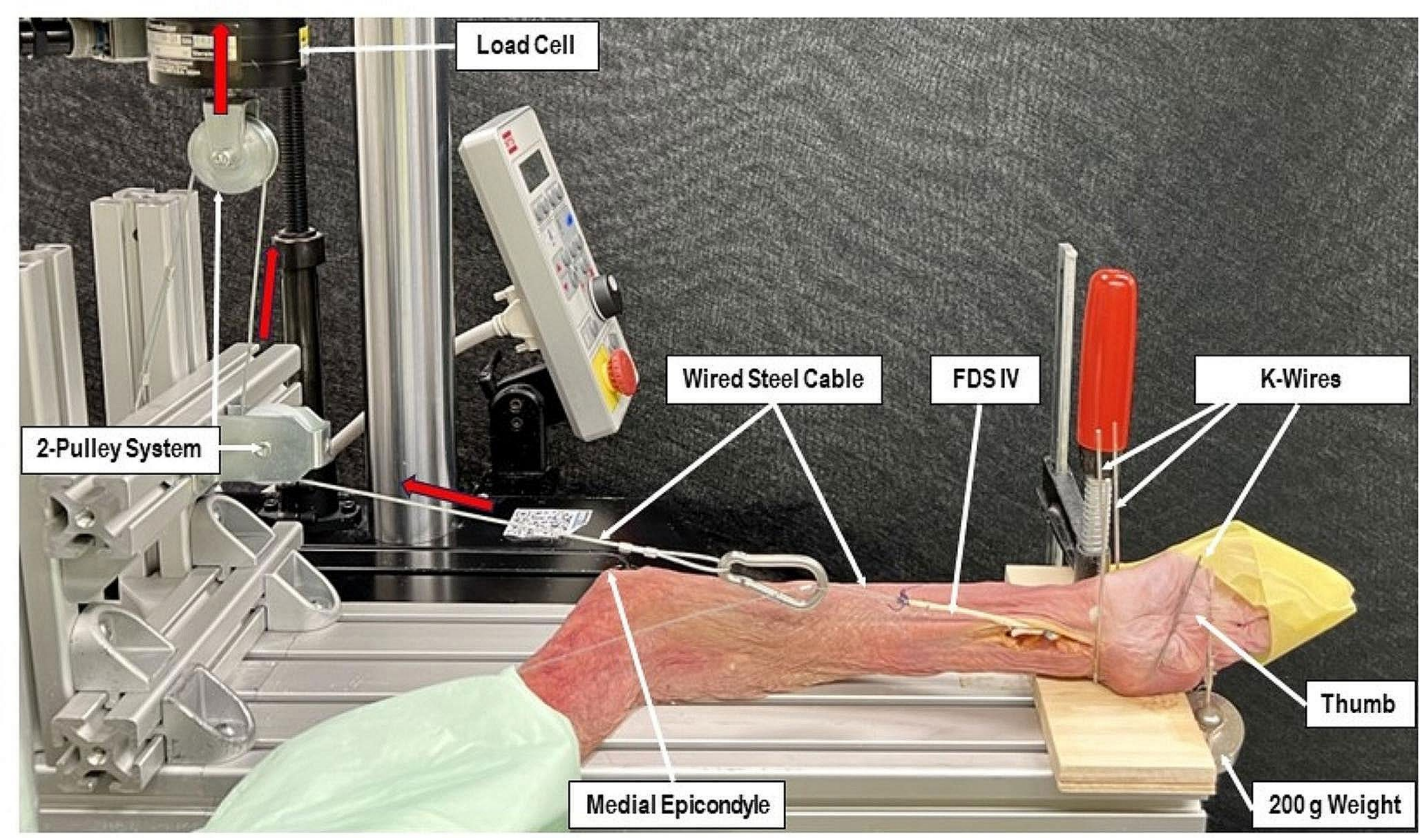
Test setup with a right specimen iterated with DC suture material and mounted for biomechanical testing. Fingers II-IV are taped in extension in order to allow free passage of the flexing thumb. FDS: superficial flexor tendon

An electrodynamic material testing machine (Acumen III, MTS Systems Corp., Eden Prairie, MN, USA) equipped with a 3 kN load cell was used for biomechanical testing (Fig. 2). The specimens were placed in supine position on a wooden board such that the thumb was passively extended via an attached 200 g weight. Additionally, an interphalangeal (IP) joint arthrodesis using a 1.4 mm Kirschner (K-) wire was performed in order to stabilize the thumb during flexion. The hand was fixed on the board with a 1.6 mm K-wire through the first metacarpal bone radial to the FPL tendon. Besides that, one 2.0 mm K-wire was placed in front of the radial wrist and two 2.0 mm K-wires were placed at its dorsum, prohibiting any kind of movement of the forearm or wrist and avoiding irritation to the tested tendons. The wooden board was then securely attached to the base of the testing machine. The looped steel cables were attached to the testing machine via a simple pulley block system transmitting vertical load to a force vector along the axis of the tendon’s physiological way.

The loading protocol aimed to mimic aggressive early postoperative rehabilitation exercises and involved flexion-extension movements of the thumb between full extension and 60° flexion, actuating the machine between 0 N and 50 N tension in load control. Each thumb underwent repetitive dynamic loading over 300 cycles at 0.2 Hz simulating one hand therapy session of 25 min. Each specimen was tested 9 times with a break of 24 h between the cyclic test sessions, simulating 9 sessions of hand therapy accounting for one series of prescribable aftercare in Switzerland. Finally, the surgical field of each specimen was covered with its subcutaneous fat tissue as well as dressings soaked with ringer solution, and left for 24 h in the refrigerator at 6° C. The soft tissue was repeatedly sprayed with physiologic ringer solution before and during all tests. After the 9^th^ session, a test to catastrophic failure was run on the 10^th^ day.

### Data acquisition & evaluation

After each cyclic test session, the distance from the proximal mark to the first knot of the side-to-side transfer (‘proximal zone’), from the first to the last knot of the transfer zone (‘intermediate zone’), and from the last knot of the transfer zone until the distal mark (‘distal zone’) was measured using a digital calliper (Futuro, Brütsch/Rüegger, Urdorf, Switzerland) with an accuracy of 0.01 mm. Finally, peak loads after testing to catastrophic failure were obtained and failure modes were evaluated by visual inspection.

Statistical evaluation was performed with SPSS software package (IBM SPSS Statistics, V27, IBM, Armonk, NY, USA). Shapiro-Wilk test was used to screen and prove normality of the data distribution. Differences in zone lengthening and its change over the cyclic test sessions were analyzed with General Linear Model Repeated Measures test. Significant differences between the study groups were identified using Paired-Samples t-tests. Hereby, the data was normalized to cycle 0 and pooled over the 9 test sessions. Level of significance was set to 0.05 for all statistical tests.

## Results

### Zone lengthening

**Fig. 3:**
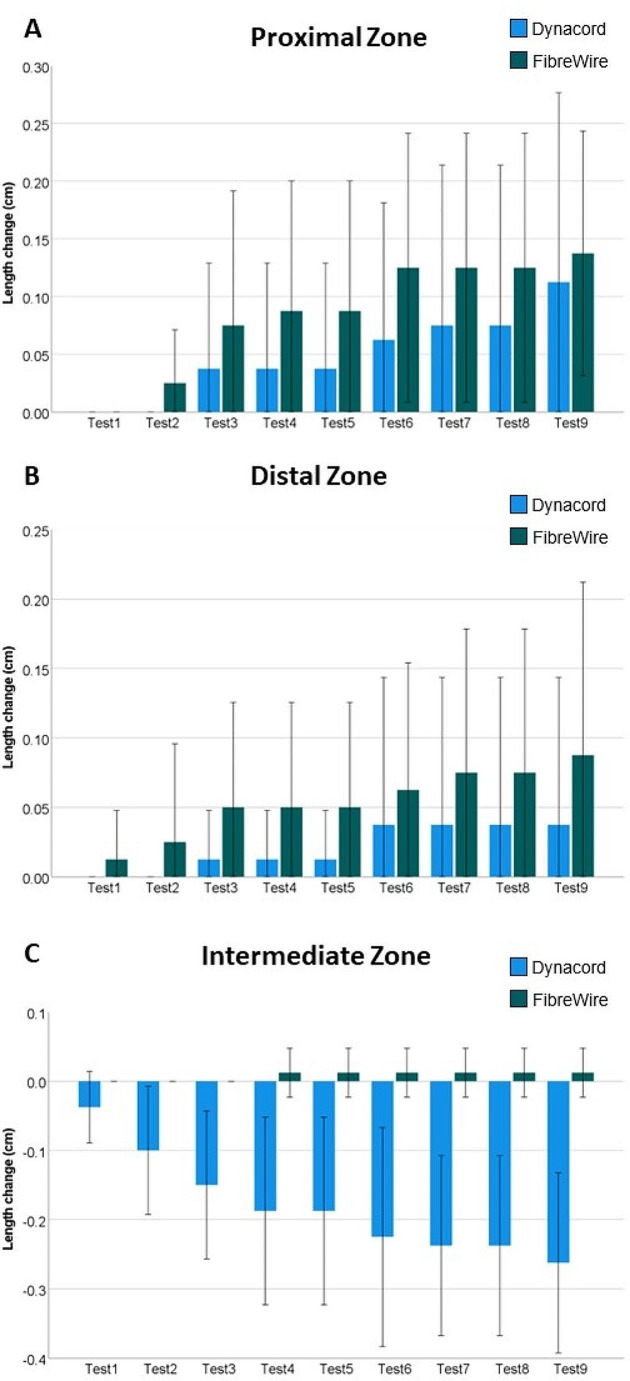
Length changes for the proximal **(A)**, distal **(B)** and intermediate **(C)** zone after each cyclic test session over 9 days (Test1 to Test9), presented for each group (Dynacord and FibreWire) separately in terms of mean value and SD

Pooled data over all 9 test sessions, normalized to the immediate postoperative status, demonstrated no significant differences in zone lengthening for FW compared to DC for the proximal (*p* = 0.396) and distal (*p* = 0.355) zone measurements (Fig. 3A & B). However, at the intermediate zone, DC was associated with significantly smaller values compared to FW (*p* < 0.001), and with a significant length shortening (mean shortening 2.6 mm, *p* < 0.001) compared to FW over the 9 test sessions, whereas FW remained without length changes (*p* = 0.351) (Fig. 3C).

### Failure modes

**Fig. 4:**
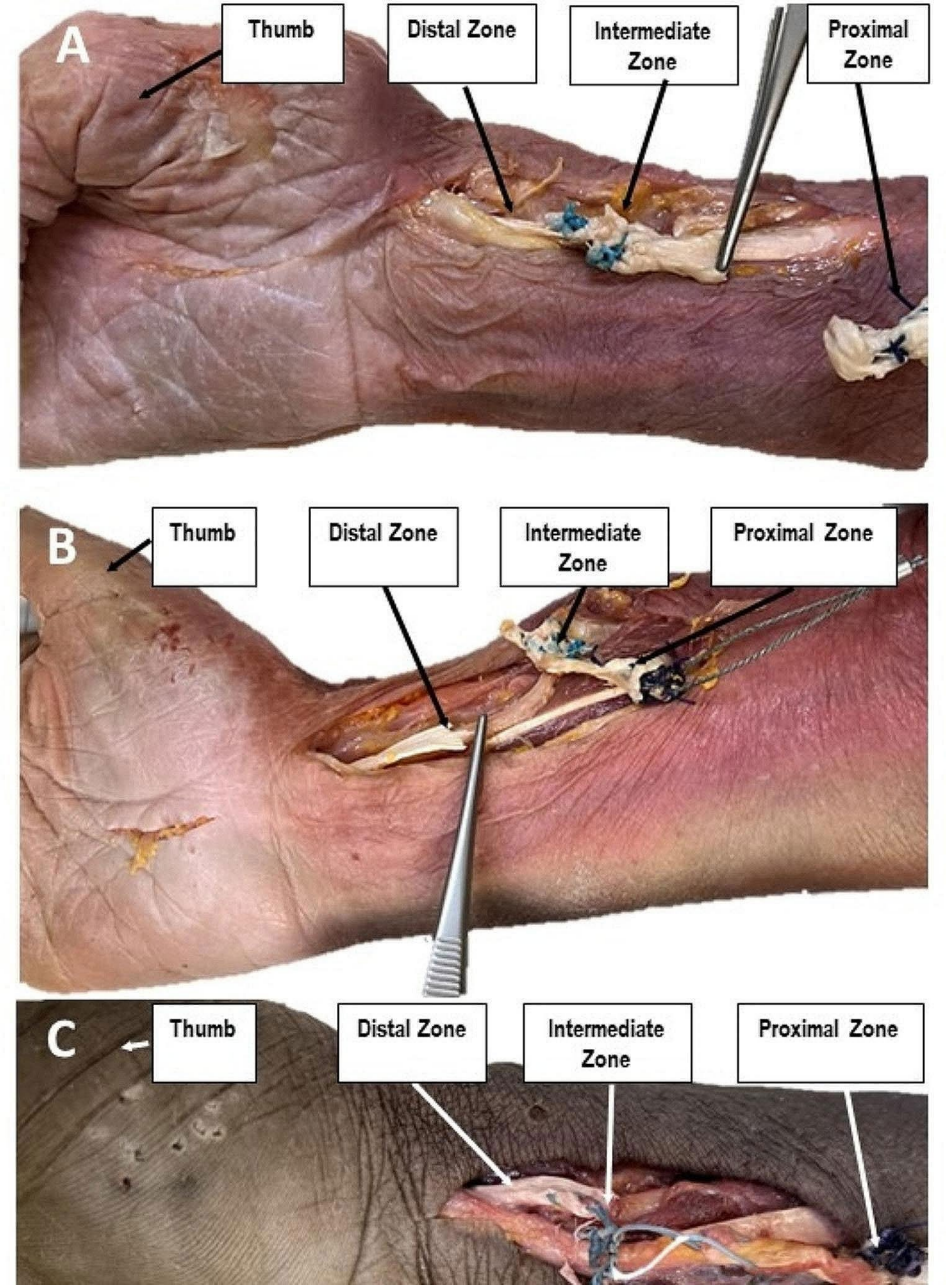
View from palmar to failures of 3 different right specimens treated with FW (**A**, **B** and **C**). **A**: Failure mode indicating tendon rupturing in the proximal zone; **B**: Failure mode indicating tendon rupturing in the distal zone; **C**: Failure mode indicating tendon rupturing in the intermediate zone

Testing to catastrophic failure on the 10^th^ day demonstrated significantly higher failure loads for FW (mean 387.0 ± 108.3 N SD) compared to DC (mean 256.3 ± 91.3 N SD), *p* = 0.011. For FW, 7 of 8 tendons ruptured proximal to the transfer zone within the healthy tendon (Fig. 4A & B) and one failure occurred in the intermediate zone (Fig. 4C). In contrast, in the DC group no failure was observed in the intermediate zone, the tendons torn within the proximal zone in 5, and within the distal zone in 3 specimens.

## Discussion

The current study compared the biomechanical competence of a novel self-tightening (DC) versus conventional high strength (FW) suture material in a human cadaveric tendon transfer model using an early aggressive rehabilitation protocol. Due to DC’s revolutionary ability to contract itself, this biomechanical investigation adds valuable knowledge to the existing literature, as DC demonstrated a significant bigger contraction within the intermediate zone compared to FW. This possibly allows an earlier more aggressive postoperative mobilisation protocol preventing soft tissue adhesions and, therefore, stiffness.

The best practice in tendon repair and transfer surgery—having requirements for an optimal technique as prerequisite as well as requirements of the used suture material— is still up for debate in the current literature [15–17]. Requirements regarding the suture material for such interventions include good gliding qualities, lack of bulkiness, as well as high tensile strength to preserve tissue approximation [18]. Although the gliding ability was not investigated in the current study, the tightening effect of DC has another advantage compared to FW. As recently proposed by van Knegsel et al. in a biomechanical study using a fatty-wet environment, only 4 knots were used in the DC group versus 7 in the FW group, leading to smaller knot stacks possibly reducing soft tissue irritation in patients [14]. The authors reported comparable knot slippage and knot security for DC with only 4 knots compared to FW with 7 knots. The practice of 7 needed knots to achieve knot security was previously described by Neuhofer et al. and later confirmed by van Knegsel et al. [19]. However, using high-strength suture material in tendon transfer surgery is not without concern and the thickness of DC and FW material cannot be ignored. In the literature, several authors reported investigations on tendon transfer surgeries using thinner sutures in cadavers. Fitzgerald et al. compared different suture thickness (Ethibond 2-0, 3-0 and 4-0) and two different suture techniques. They reported no significant differences in the yield force for each suture and observed that 6 out of 8 specimens with the 4-0 and 2 out of 8 with the 3-0 failed due to the suture material, in contrast to the thicker 2-0 suture which could withstand the loads. Considering the yield force over 100 N for all tested sutures, the authors prefer using a 3-0 suture over a 2-0 suture due to the thinner knot bulk [20]. Gillis et al. biomechanically tested a new 2-0 nonabsorbable meshed suture construct (Ethibond Excel 2-0). However, in their investigation the meshed suture needed only 3 knots [15]. Having this in mind, the size of the DC’s knot stack used in the current study might be bigger, however, comparing 3 versus 4 knots might not result in a clinically relevant difference. Furthermore, in contrast to the results by Gillis et al., the DC’s 4 knots used in the current study proved knot security in a biomechanical investigation as previously reported by van Knegsel et al. [14]. Nevertheless, in the forearm region—in contrast to the hand—bigger knot stacks might not be disturbing due to the soft tissue around it. One possibility to overcome these downsides might be to cover the knot stack with the tendon sheet itself by pulling it down to the tendon. Future clinical studies need to evaluate whether the knot stack is disturbing at all in the forearm region. Besides that, the novel dynamic suture DC should be produced thinner in order to be placed in the distal forearm or even in the hand without irritating the soft tissues. A looped suture would be indispensable for flexor tendons—e.g., in acute lacerations.

Main requirements for the technical part of the transfer or tendon graft reconstructions are a strong interweaving zone to withstand the high mechanical forces, and a slimmest possible tendon overlay zone. Pulvertaft et al. described a technique for interweaving the donor and recipient tendon several times into another to increase the biomechanical strength [21]. Modifications of this Pulvertaft technique (PT) have later been introduced, describing minimized bulkiness of the transfer zone [22–25]. Nevertheless, a series of recent investigations has shown that a side-to-side repair with only a single weave—as used in the current study—is biomechanically stronger compared to the PT technique [26–29]. Fridén and Reinholdt described a side-to-side repair [9] which was biomechanically evaluated later on by Brown et al. who found it stronger (mean max load to failure 182 N) as compared to the PT technique (mean max load to failure 92 N) [13]. The authors calculated a minimum overlap of the tendons of at least 30 mm for the site-to-site repair technique with an Ethibond 3-0 suture material. In the current investigation biomechanical testing was done with a 11 mm tendon overlap. This length was chosen due to fact that one of the specimens had a very short tendinous portion of the FDS chord. Therefore, an overlap of only 11 mm could be achieved due to the suspension on the testing machine. To standardize the surgical technique, all specimens consequently received a tendon overlap of 11 mm. Nevertheless, the results of the current study suggest that 11 mm overlap might be sufficient when high-strength suture material is used in tendon transfer surgery as only one failure occurred in the intermediate zone in the FW group and none in the DC group during load to failure testing. Furthermore, tendons in both groups ruptured at a mean load of 387 N for FW and 256 N for DC, which is far stronger than the previously reported 92 N in PT and 182 N in side-to-side repair [13], and the 100 N reported by Fitzgerald et al. [20]. It is therefore hypothesized that the postoperative motion protocol after tendon transfer or reconstruction surgery might allow finger movements against resistance when high strength sutures ae used. However, the amount must be defined in future biomechanical research as the current study only evaluated flexion against gravity with an additional 200 g weight. Furthermore, using stronger suture material—especially in short and flimsy tendons—may allow for a shorter tendon overlap leading to less bulkiness in the site-to-site repair which should be evaluated with different suture materials in further biomechanical and clinical studies.

The current study evaluated the FDS-IV to FPL tendon transfer. Nevertheless, the results may be transferred to other transfer or even tendon reconstructions with tendon grafts in the upper extremity apart from the fingers and the hand. This transfer was chosen for the current study as the FDS-IV tendon has an adequate length to be sutured to the FPL tendon as well as to be attached proximally to the testing machine. Furthermore, the surgical technique, the biomechanical testing and the procedure were standardizable. However, the FDS-IV to FPL tendon transfer is controversy debated in the current literature [11]. On one hand, flexion contractures of the proximal interphalangeal joint (PIP) of the ring finger as well as insufficient range of motion of the interphalangeal joint (IP) or a tendon-vaginosis are described [11, 30, 31]. Moreover, there are clinical reports with outstanding results after this procedure with no impairment of movement of the ring finger nor the thumb, and with an overall satisfying grip strength [32, 33]. The reason to the different results might be the use of non-standardized surgical techniques. Nevertheless, attention must be paid during FDS-IV tendon harvesting. Posner et al. advocated that the donor tendon should be harvested between the A1 and A2 pulley with the PIP joint in slight flexion. This way the vincula vessels are preserved and a hyperextension in the PIP joint is avoided [32].

There is no consensus in the literature regarding the optimal postoperative protocol, the duration of immobilization and the timing of rehabilitation vary among different studies [9–11], however, there is a trend to early mobilization to decrease the risk of soft tissue adhesions, which presents a common postoperative complication in tendon transfer surgery [34]. The use of the tenodesis effect during postoperative rehabilitation is one attempt to lower these complications and an even more aggressive rehabilitation protocol possibly with early movements against slight resistance would represent a further increase of this approach.

After a surgical intervention, Swiss hand surgeons can prescribe 9 hand therapy sessions. Therefore, the current study used a test setup with 300 cycles—simulating one hand therapy session of 25 min—for 9 days in a row with time intervals of 24 h in between, rendering the used test protocol as clinically relevant.

During the tests to catastrophic failure, a significantly higher load at failure was found in the FW group. However, main failure mode for FW was tendon rupture in the proximal zone in all but one specimen with only one failure within the intermediate zone and one in the distal zone. In contrast, tendon rupture for DC occurred in the proximal zone of 5 specimens, for none specimen in the intermediate zone, and for 3 specimens in the distal zone, rendering the used surgical technique and the interweaving zone as biomechanically stronger compared to the intact tendon in the proximal and distal zone. Therefore, the significant difference between DC and FW in load at failure cannot be just considered as resulting from the differences between the two investigated sutures and other reasons such as tendon thickness due to left-right dominance or preexisting tendon variations may play a role. The reason of the one failure in the intermediate zone might be explained due to poor surgical technique—the stitches could have been made between the same or adjacent fibres of the tendon and not in different fibres as required for higher stability, which could have possibly reduced the resistance to high loads of the intermediate zone ultimately leading to failure.

### Methodological considerations

Limitations of the current study include the employment of human cadaveric specimens that does not perfectly mirror living tissue’s healing, soft tisse and tendon quality as well as mechanical behaviour. Furthermore, only a limited number of specimens was tested, which restricts generalization of the above-mentioned findings. Nonetheless, the results are deemed adequate as they show notable variations between the groups. The tendon transfer was performed at the level of the distal forearm which might contrasts clinical scenarios, where the tendon rupture is located more distally. However, to gain a clear view of the operating field for later measurements, sufficient space for the transfer and the marking sutures was needed. Furthermore, an interphalangeal joint arthrodesis was performed to stabilise the thumb. This was done as one specimen demonstrated hypermobility in the IP joint and contrasts with clinical practice. The arthrodesis changed the mean gliding distance of the FPL by 1.6 mm (range 1–2 mm) at the level of the scaphoid’s tuberculum in all specimens. Finally, all measurements were performed manually using a calliper, which might have biased the results. Yet, the measurements were performed by a single researcher using a standardised technique.

The strengths of the current investigation were the uniformity of all surgical procedures done by one experienced hand surgeon with the assistance of an orthopaedic surgeon. Furthermore, a reliable assignment to the two study groups was ensured using paired cadaveric specimens tested with a standardized test setup and protocol.

Future research should focus on gliding abilities of the investigated high strength suture material as well as possible interfering reactions to the surrounded soft tissues, which might be less in the forearm region compared to the hand. Furthermore, the amount of finger movement against resistance should be evaluated applying more than 200 g weight. The tendon interweaving zone of 11 mm might be beneficial if only short tendons are available for transfer surgery (e.g., after trauma) and should be further evaluated in clinical and biomechanical investigations, especially using the evaluated high-strength sutures. In this context, future research using the Fridén technique with two times tendon interweaving using a tendon graft should be explored biomechanically. Finally, DC demonstrated promising results in this biomechanical evaluation and should therefore be evaluated in future clinical studies.

## Conclusion

From a biomechanical perspective, DC preserved or even increased tissue approximation, and might be considered as a valid alternative to conventional high-strength sutures in tendon transfer surgery. DC might allow for a shorter interweaving zone and a more aggressive early postoperative rehabilitation program, possibly avoiding commonly observed postoperative soft tissue adhesions and stiffness.

## Electronic supplementary material

Below is the link to the electronic supplementary material.

Supplementary Material 1
