# Correction: New dynamic suture material for tendon transfer surgeries in the upper extremity – a biomechanical comparative analysis

**DOI:** 10.1007/s00402-024-05450-y

**Published:** 2024-09-06

**Authors:** Tatjana Pastor, Ivan Zderic, Mehar Dhillon, Boyko Gueorguiev, R. Geoff Richards, Torsten Pastor, Esther Vögelin

**Affiliations:** 1 AO Research Institute Davos, Davos, 7270 Switzerland; 2 Department for Plastic and Hand Surgery, Inselspital University Hospital Bern, University of Bern, Bern, Switzerland; 3 Department of Orthopaedic and Trauma Surgery, Lucerne Cantonal Hospital, Lucerne, Switzerland

**Archives of Orthopaedic and Trauma Surgery (2024) 144:2905–2914**

10.1007/s00402-024-05322-5

In the original version of this article, the ORCiD for Ivan Zderic, Boyko Gueorguiev, R. Geoff Richards and Torsten Pastor were inadvertently omitted and should have been

Ivan Zderic 0000-0003-0484-887X

Boyko Gueorguiev 0000-0001-9795-115X

R. Geoff Richards 0000-0002-7778-2480.

Torsten Pastor 0000-0003-4357-236X.

And, Fig. 4 was not fully displayed. The Fig. 4 should have appeared as shown below.

**Fig. 4:**
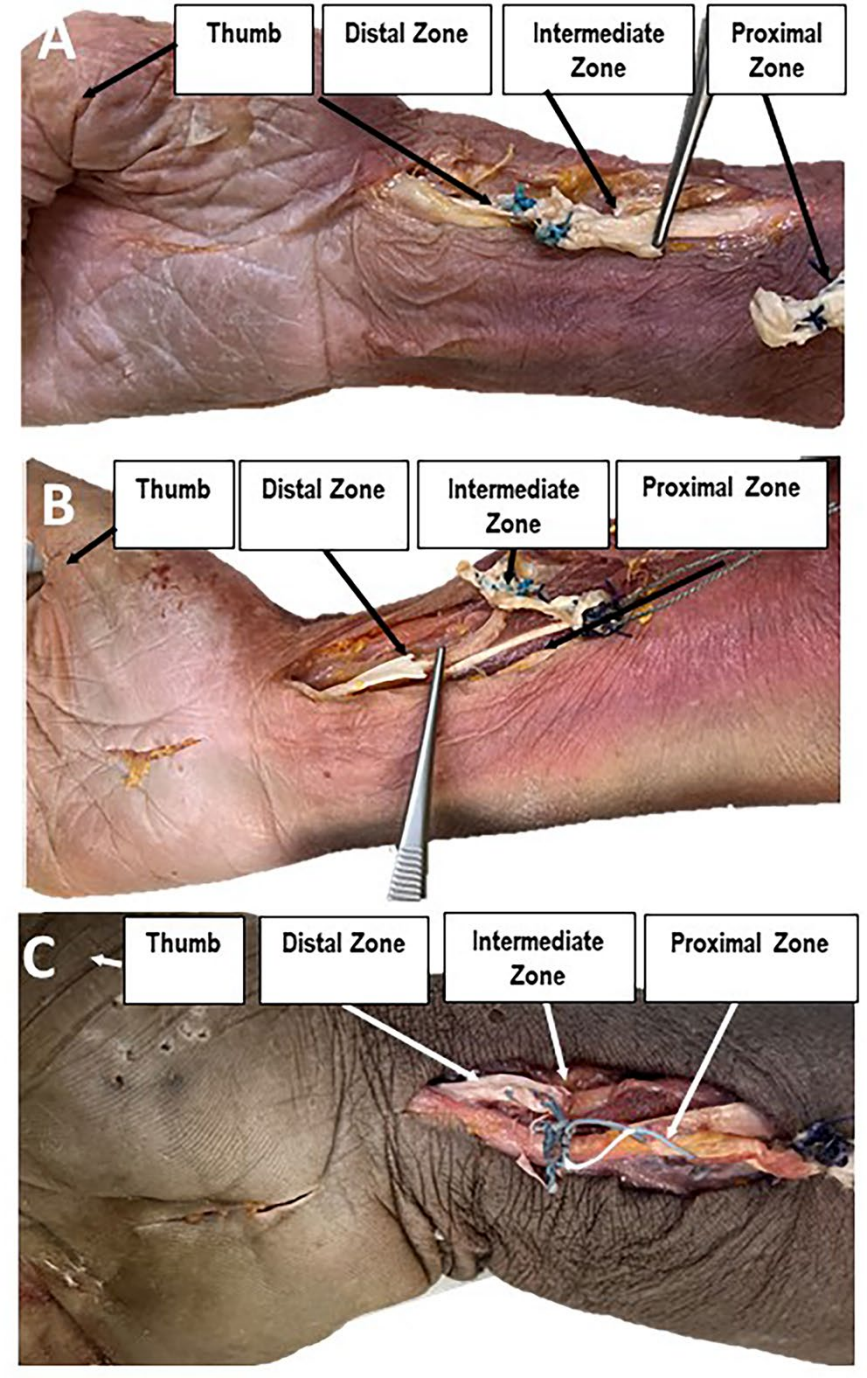
View from palmar to failures of 3 different right specimens treated with FW (**A**, **B** and **C**). **A**: Failure mode indicating tendon rupturing in the proximal zone; **B**: Failure mode indicating tendon rupturing in the distal zone; **C**: Failure mode indicating tendon rupturing in the intermediate zone

