# Supplementary material for: New dynamic suture material for tendon transfer surgeries in the upper extremity – a biomechanical comparative analysis
Source: Arch Orthop Trauma Surg. 2024 May 2;144(6):2905–14. doi: 10.1007/s00402-024-05322-5 (PMC11211109; doi:10.1007/s00402-024-05322-5)

---=P**AR**,;,.**C**O**HIVES O**T**f__**

Orthopaedic and

Trauma Surgery

rn::.1 €1 **Springer**

the language of science

Conflict of Interest and Disclosure Form

It is the policy of the Journal *Archives of Orthopaedic and Trauma Surgery* to ensure balance, independence, objectivity, and scientific rigor in the Journal. All authors are expected to disclose to the readers any real or apparent conflict(s) of interest that may have a direct bearing on the subject matter of the article. This pertains to relationships with pharmaceutical companies, biomedical device manufacturers or other corporation whose products or services may be related to the subject matter of the article or who have sponsored the study.

The intent of the policy is not to prevent authors with a potential conflict of interest from publication. It is merely intended that any potential conflict should be identified openly so that the readers may form their own judgements about the article with the full disclosure of the facts. It is for the readers to determine whether the authors' outside interest may reflect a possible bias in either the exposition of the conclusions presented.

The senior and the corresponding author will complete and submit this form to the Springer Journal's Editorial Office, Carsten Brall (Mr) on behalf of all authors listed below

.

*Article Title*

*Authors*

*New dynamic suture material for tendon repair and transfer surgeries*

*in the upper extremity – a biomechanical comparative analysis*

*Tatjana Pastor, Ivan Zderic, Mehar Dillon, Boyko Gueorguiev ,*

*R. Geoff Richards, Torsten Pastor,, Esther Vögelin*


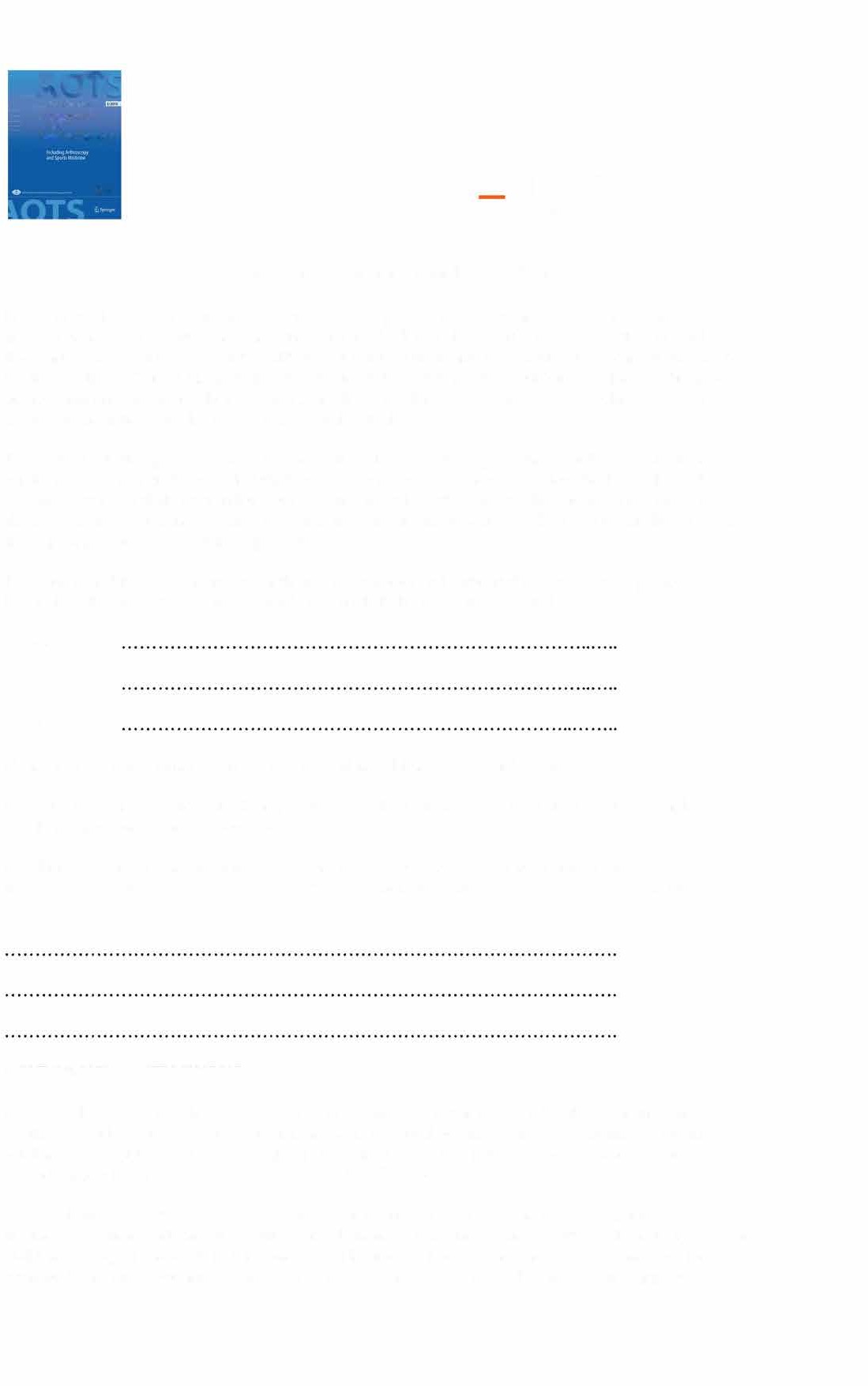
Please note that a conflict of interest statement is published with each paper.

I certify that there is no actual or potential conflict of interest in relation to this article. If any conflict exists, please define hereafter:

*Conflict (if none, "None" or describe financial interest/arrangement with one or more organizations that could be perceived as a real or apparent conflict of interest in the context of the subject of this article):*

*None*

***CONDITIONS OF SUBMISSION***

ORIGINALITY: Each author warrants that his or her submission to the Work is original and that he or she has full power to enter into this agreement. Neither this work nor a similar work has been published or submitted, nor shall it be submitted for publication elsewhere while under consideration by the *Archives of Orthopaedic and Trauma Surgery.*

AUTHORSHIP RESPONSIBILITY: Each author certifies that he or she has participated sufficiently in the conception and design of this work, intellectual content, the analysis of data, if applicable, and the writing of the work to take responsibility for the integrity and accuracy of the data. Each has reviewed the final version of the work, believes it represents valid work, and approves it for


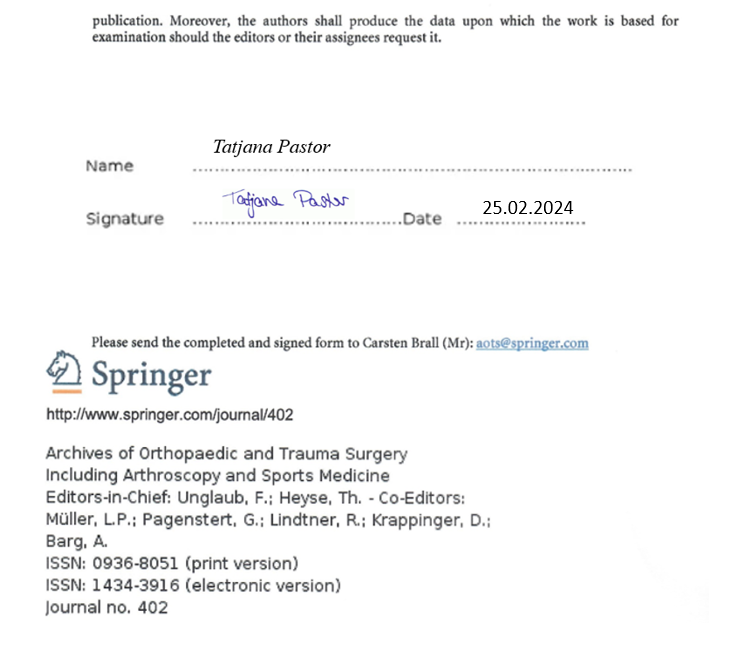

Supplement: Supplementary file 1 — Supplementary Material 1 [file 402_2024_5322_MOESM1_ESM.docx]
